# Real-time navigation of mecanum wheel-based mobile robot in a dynamic environment

**DOI:** 10.1016/j.heliyon.2024.e26829

**Published:** 2024-03-06

**Authors:** Muhammad Umair Shafiq, Abid Imran, Sajjad Maznoor, Afraz Hussain Majeed, Bilal Ahmed, Ilyas Khan, Abdullah Mohamed

**Affiliations:** a Faculty of Mechanical Engineering, GIK Institute of Engineering Sciences and Technology, Swabi 23640, Pakistan; b Research Institute of Engineerig and Technology, Hanyang University (ERICA), Ansan 15588, South Korea; c Department of Electrical Engineering, Mirpur University of Science and Technology, 1025, Mirpur AJK, Pakistan; d School of Energy and Power Engineering, Jiangsu University, Zhenjiang 212013, China; e Department of Electrical Engineering, University of Poonch Rawalakoot, Pakistan; f Department of Mathematics, Saveetha School of Engineering, SIMATS, Chennai, Tamil Nadu, India; g Department of Mathematics, College of Science Al-Zulfi, Majmaah University, Al-Majmaah 11952, Saudi Arabia; h Research Centre, Future University in Egypt, New Cairo 11835, Egypt

**Keywords:** Mobile robot with mecanum wheels, Path planning, Dynamic environment, $A^{⁎}$ algorithm, Velocity obstacle

## Abstract

Path planning and control of a mobile robot, in a dynamic environment, has been an important research topic for many years. In this paper an algorithm for autonomous motion of a mobile robot is proposed, with mecanum wheels, to reach a goal while avoiding obstacles through the shortest path in a dynamic environment. The proposed method uses a hybrid $A^{⁎}$ and a velocity obstacle algorithms for path planning and obstacle avoidance. The $A^{⁎}$ algorithm is implemented to explore the shortest path from starting position to the goal while avoiding all the static obstacles. However, in real time applications the dynamic obstacles need to be avoided, therefore, for such a case velocity obstacle algorithm is unified with the $A^{⁎}$ algorithm. Initially, the proposed algorithm is verified through simulations. Then it is implemented using experimental setup in real time environment using single and multiple static obstacles as well as on a dynamic obstacle. It can be observed that the robot reaches the goal, effectively by avoiding static and dynamic obstacles. Moreover, the performance of the proposed work is evaluated through qualitative comparison between proposed method and recently published work, showing that the proposed algorithm is gives better features than existing work. In the end, the possible application of mobile robot having mecanum wheels with proposed path planning method is also given in the paper.

## 1 Introduction

Autonomous path planning and navigation is one of important research field for mobile robots. Due to their various abilities and working efficiency, mobile robots can replace humans in a variety of industries. Patrolling, monitoring, guidance, emergency rescue operations, industrial automation, construction, entertainment, transportation, and medical care, are among the many industrial and nonindustrial applications a mobiles robot can perform [1]. In all these applications, to achieve better efficiency it is desired to move mobile robots autonomously without the involvement of human operators. This can be achieved with the help of better design, good choice of specifications and an efficient control algorithm [2].

A wheeled mobile robot (WMR) is extremely important for tracking and avoidance of obstacle in an unstructured environment. A lot of work has been done on the motion planning of mobile robots in past few decades. An algorithm for localization and control of differential drive robot is proposed in [3] by implementing the closed loop control, to make the robot to follow the desire obstacle free path in indoor static environment. In [4], design of differential drive mobile robot, and its kinematic modeling is given. Path planning, and localization of differential drive mobile robot can be achieved by using odometry [5], by using fuzzy logic to control the mobile robot. In [6] the authors proposed the position and velocity control on two-wheel differential drive robot, implemented to reduce the position error from one set point to other desired point. The kinematic model, using transverse feedback linearization with dynamic extension, for control of a car like robot is proposed in [7]. In [8], the kinematic modeling for the control of non-holonomic car like robot is proposed by considering the motion of the wheel in both direction such that wheel can roll without slipping in any direction. Firstly the model of single wheel was analyzed and then it was implemented for multiple robots. A reciprocal collision avoidance system for multiple robots of non-holonomic constraints is proposed in [9]. The kinematic modeling of mobile robot is achieve considering it non holonomic car like robot in which a rear wheel is fixed and with the steerable front wheel [10]. In [11] the authors studied the car-like robot its kinematic model, trajectory tracking and control problems to get an optimal analytical solution that ensures the tracking error's global exponential stability. Position, velocity and acceleration errors are reduced. Subject to the kinematic model of a rear-wheel car-like robot to convert the data, the input-output linearization approach is used. Converting a nonlinear issue to a linear one the analytical solution is derived using the variational technique.

Omnidirectional mobile robots are special kind of mobile robots which can move in any direction, due reason, they can easily skid instead of turning. Kinematic model of omnidirectional wheel provides the analytical control of mobile robot [12]. The geometry and kinematics of mecanum wheel can be used to find out the relationship between velocity of the robot and the velocity of each wheel, as given in [13]. In [14], the authors proposed the mathematical modeling of four mecanum wheel mobile robot along with the calculated both forward and inverse kinematics of omnidirectional mecanum wheel. It can be observed that eight different motions are obtained without changing the orientation of robot. A kinematics and dynamics model of omni-directional mecanum wheel is given in [15], in order to achieve the autonomous motion of the vehicle in congested environment such as in office, factory and other such type of environment. In [16] the authors proposed the modeling and kinematics simulation of RecurDyn, where the mecanum wheel platform is used for wheelchair. The kinematics of the RecurDyn is obtained to move the wheelchair in any direction in closed loop system.

A velocity obstacle algorithm can be used, for path planning of mobile robot to avoid both dynamic and static (dynamic obstacle with zero velocity) obstacles [17], [18]. Collision and velocity obstacle cone need to be calculated in order to implement the velocity obstacle algorithm. Subsequently, the reachable velocities can be calculated avoidance using the velocity obstacle information. In [19] the authors proposed a green-lined velocity obstacle algorithm, which was initially used only for holonomic systems, and modified the algorithm for the non-holonomic systems such as car like robot. The VO works very well in simulation environment, however, in real time environment, due to the uncertainty of sensors, there will always be a collision risk. In order to get real time implementation of the velocity obstacle algorithm a modified velocity obstacle algorithm was proposed in [20] which is called safety velocity obstacle. In this method, velocity obstacle is calculated for some specific interval of time. After every interval of time, distance of robot is calculated from the velocity obstacle cone. If the distance is greater than the threshold distance then the VO is set for maximum distance. Maximum distance can be calculated by multiplying the maximum velocity with the sampling time. This method decreases risk of collision despite of uncertainty if sensors in real time environment.

In [21] the velocity obstacle algorithm is modified for non-holonomic vehicle, in such a way that constraints such as, following a specific path or the velocity of obstacle are no more required. This algorithm is implemented on non-holonomic constraints with limiting turning rate in [22]. In [23] authors introduced a new method to navigate in dynamic environment which is called reciprocal velocity obstacle. This is an extended work of velocity obstacle which is implanted on multi agents. It can be implemented on both static and dynamic environment. According to Reciprocal velocity obstacle (RVO), position and velocity of each agent is known or measurable. Each agent makes its own collision avoidance cone to avoid other agents as well as any other moving obstacles. In this paper, implementation of RVO is done in 2D agents. This technique is also feasible for high-speed agents. In [24], the authors proposed the velocity obstacle algorithm for high-speed obstacle in such a way that the speed of moving obstacle is higher than the speed of the robot. A two period velocity obstacle method is proposed where one period predicts the collision within limited time horizon and the second period is activated after that time horizon. Second period is activated only when the speed of obstacle is higher than the speed of mobile robot. A new velocity obstacle is constructed after each step which can predict the collision beyond the time horizon of the existing VO based method. A modified velocity obstacle algorithm for high-speed obstacle via maximum speed aware velocity obstacle algorithm is proposed in [25]. In real time application all robots can't move freely in any direction due to non-holonomic constraint e.g., car like robot. The field of view of robot is limited and robot can only avoid obstacle within its perception range. This method has also incorporated this type of constraints. This is a two-window velocity obstacle method where one window will be activated with in time horizon and other window will be activated after this time horizon.

In [26], the authors used the velocity obstacle algorithm for the collision avoidance of unmanned surface vehicle. The results of velocity obstacle algorithm are validated experimentally by the designing of unmanned surface vehicle. Various algorithms have been designed and modified for collision free path. A modified three-dimensional velocity obstacle algorithm was proposed and implemented on and unmanned aerial vehicle (UAV) in [27]. To prevent these accidents monitoring is done through underwater vehicle, which is very important for maritime inspection. A time based nonlinear velocity obstacle algorithm was proposed in [28] for collision detection in ships.

Various path planning techniques are implemented on the mobile robot over the years. Choices of path planning techniques are based on the applications of mobile robots. To the best of authors knowledge, to avoid the dynamic obstacles, velocity obstacles algorithm is successfully implemented on ROV and UAV, however, it is not very common in mobile robots. The main contributions of the paper are as follows;

1. A Lidar sensor is used to calculate the size, position and velocity vector for the dynamic environment.
2. An A* algorithm is used to find the static obstacles free shortest path between start point and goal point.
3. Integration and complementation of velocity obstacles method in real time is done to avoid the dynamic obstacles.
4. Possible application of the proposed setup to assist the visually impaired people in structured environment is also given.

The application of the robot as an assistive device will help the visually impaired people to move around in dependent in daily life movement. The robot has the capability to avoid the multiple static and dynamic obstacles in open environment.

The paper is further organized as follows; in Section 2 velocity kinematics of mecanum wheels based mobile robot is given for various types of motion. The proposed path planing algorithm along with the details of the A* algorithm and velocity obstacle is also given in this section. In Section 3 the results achieved through the simulations and experiments are presented and discussed for validation of proposed algorithm. In Section 4 the paper is concluded and future work is also proposed. The possible application of mobile robot having mecanum wheel with proposed path planning method as assistive device for visually impaired people is also given in this section.

## 2 Material and methods

### 2.1 Mobile robot kinematics

**Figure 1:**
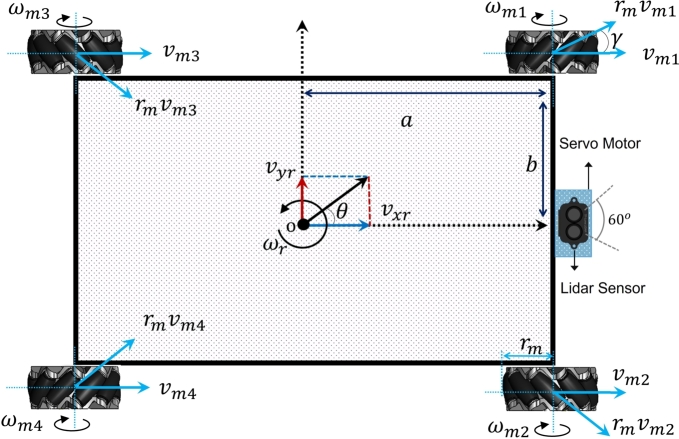
Free body diagram of mobile robot with mecanum wheels.

In this research an omnidirection robot is used, which has capability to move in any direction. It is holonomic robot with four special wheels i.e. mecanum wheel system, each driven by a separate stepper motor. For such a robot, the number of controlled degree of freedoms are equal to total number of degree of freedoms of the robot. It can move in any direction, on plane surface, due to its free rotation rollers place on the wheel surface at $45^{\circ }$ degree. In Fig. 1 a mobile robot model with mecanum wheel is given with coordinate system attached at the center of the wheel hub the unit axis is denoted by $\left(X_{w},Y_{w}\right)$. The robot position and orientation is denoted as $x_{r},y_{r},\theta_{r}$. The linear velocity of robot is $\left(v_{xr},v_{yr}\right)$ and $\omega_{r}$ is its angular velocity, while $\omega_{mi}$ is angular velocity of $i^{th}$ wheel and $v_{mi}$ donated the linear wheel velocity. The angle between the free sliding roller axis and the wheel hub axis can be either positive $45^{\circ }$ or negative $45^{\circ }$ depending on whether the wheel is left or right.

Let, $v_{driver}$ and $v_{slider}$ are two separate velocity components, where linear velocity of the wheel is $v_{driver}$, whereas the linear velocity of the free sliding roller is $v_{slider}$. The relation between center velocity $\left(v_{xr},v_{yr}\right)$ and $v_{driver}$ and $v_{slideris}$ is given as;

(1) $$\left[\begin{array}{c} v_{xr}\\ v_{yr} \end{array}\right]=v_{driver}\left[\begin{array}{c} 1\\ 0 \end{array}\right]+v_{slider}\left[\begin{array}{c} -sin\gamma \\ cos\gamma \end{array}\right]$$

While the relation between angular velocity of the $\omega_{m}$ and $v_{driver}$ is given as follows;

(2) $$v_{driver}=\omega_{m}Xr_{m}$$

Combining the Eq. (1) and Eq. (2) in more compact form for $i_{th}$ wheel we get;

(3) $$\left[\begin{array}{c} v_{ixr}\\ v_{iyr} \end{array}\right]=\left[\begin{array}{c} r_{i}\;\;\;-sin\gamma_{i}\\ 0\;\;\;\;\;cos\gamma_{i} \end{array}\right]\left[\begin{array}{c} \omega_{ir}\\ v_{ir} \end{array}\right]{=}_{pi}^{wi}T\left[\begin{array}{c} \omega_{ir}\\ v_{ir} \end{array}\right]$$

Transformation matrix from wheel coordinate system to mobile coordinate is given as;

(4) TpiR=[cos(θr)sin(θr)−sin(θr)cos(θr)]

While the component of linear velocity of mobile from each wheel velocity can be written as;

(5) $$\left[\begin{array}{c} v_{ixr}\\ v_{iyr} \end{array}\right]=\left[\begin{array}{c} 1\;\;\;0\;\;\;-a_{i}\\ 0\;\;\;1\;\;\;\;b_{i} \end{array}\right]=\left[\begin{array}{c} v_{xr}\\ v_{yr}\\ \omega_{r} \end{array}\right]=T\left[\begin{array}{c} v_{xr}\\ v_{yr}\\ \omega_{r} \end{array}\right]$$

Combining Eq. (3), Eq. (4) and Eq. (5) we get;

(6) $$\left[\begin{array}{c} \omega_{mi}\\ v_{mi} \end{array}\right]=\left[\begin{array}{c} \frac{1}{r_{mi}}\;\;\frac{tan(\gamma )}{r_{mi}}\;\;\frac{-a_{i}+b_{i}\tan(y)}{r_{m}i}\\ 0\;\;\;\;\;\frac{1}{cos(\gamma )}\;\;\frac{b_{i}}{cos(\gamma )} \end{array}\right]\left[\begin{array}{c} v_{xr}\\ v_{yr}\\ \omega_{r} \end{array}\right]$$

The independent variables $v_{mi}$ and $\omega_{mi}$ are coupled if there is no wheel slipping on the ground, the first row from Eq. (6) is taken to from Jacobian matrix of inverse transformation which transform mobile robot task space velocity to its joint velocity as follows;

(7) $$\left[\begin{array}{c} \omega_{m1}\\ \omega_{m2}\\ \omega_{m3}\\ \omega_{m4} \end{array}\right]=\left[\begin{array}{c} \frac{1}{r_{mi}}\;\;\frac{tan(\gamma_{i})}{r_{mi}}\;\;\frac{-a_{i}+b_{i}\tan(y)}{r_{m}i}\\ \frac{1}{r_{mi}}\;\;\frac{tan(\gamma_{i})}{r_{mi}}\;\;\frac{-a_{i}+b_{i}\tan(y)}{r_{m}i}\\ \frac{1}{r_{mi}}\;\;\frac{tan(\gamma_{i})}{r_{mi}}\;\;\frac{-a_{i}+b_{i}\tan(y)}{r_{m}i}\\ \frac{1}{r_{mi}}\;\;\frac{tan(\gamma_{i})}{r_{mi}}\;\;\frac{-a_{i}+b_{i}\tan(y)}{r_{m}i} \end{array}\right]\left[\begin{array}{c} v_{xr}\\ v_{yr}\\ \omega_{r} \end{array}\right]$$

Where for wheel 1 and wheel 4, *γ*=-$45^{\circ }$ while for wheel 2 and wheel 3, $\gamma =45^{\circ }$. The Eq. (7) becomes

(8) $$\left[\begin{array}{c} \omega_{m1}\\ \omega_{m2}\\ \omega_{m3}\\ \omega_{m4} \end{array}\right]=\frac{1}{r_{mi}}\left[\begin{array}{c} 1\;\;-1\;\;-a-b\\ 1\;\;1\;\;a+b\\ 1\;\;-1\;\;a+b\\ 1\;\;1\;\;-a-b \end{array}\right]\left[\begin{array}{c} v_{xr}\\ v_{yr}\\ \omega_{r} \end{array}\right]$$

**Figure 2:**
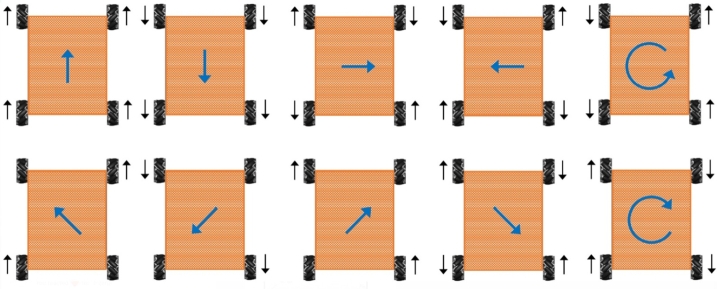
Direction of mobile robot motion based on mecanum various velocities of the wheels.

Eq. (8) describes the relation of angular velocity of each wheel with the velocity of mobile robot. Based on these velocities mobile robot moves in any direction. The direction of mobile robot's motion based on the mecanum wheel motion is shown in Fig. 2.

The following forward kinematic equation can be obtained by taking inverse matrix in Eq. (8), using left inverse matrix;

(9) $$\left[\begin{array}{c} v_{xr}\\ v_{yr}\\ \omega_{r} \end{array}\right]=\frac{1}{4r_{m}}\left[\begin{array}{c} 1\;\;\;1\;\;\;1\;\;\;1\\ -1\;\;1\;\;\;-1\;\;1\\ 1\;\;\;1\;\;\;1\;\;\;1\\ \frac{-1}{a+b}\;\;\;\frac{1}{a+b}\;\;\;\frac{1}{a+b}\;\;\;\frac{-1}{a+b} \end{array}\right]\left[\begin{array}{c} \omega_{m1}\\ \omega_{m2}\\ \omega_{m3}\\ \omega_{m4} \end{array}\right]$$

Eq. (9) describes the relation of mobile robot's velocity with the velocity of each wheel. Where $r_{m}$ is the radius of mecanum wheel and a and b describes the length and width of the mobile robot. The Eqs. (8) and (9) describe the relation between mobile robot's velocity and rotational velocity of each wheel. In this research, rpm required for each motor is calculated using Eq. (8) radius of the mecanum wheel is 0.96 m. Length and width of mobile robots is 0.38 m and 0.2 m respectively. If $v_{xr}$ and $v_{yr}$ are the velocity components of mobile robots which are user defined. By putting all the parameters one can calculate the required rpm of each wheel for desired robot's linear and angular velocities.

### 2.2 Path planing algorithm

In autonomous navigation, path planning is divided into global path planning and local path planning. In the global path planning technique all the information of environment is fed in mobile robot before moving the robot. This technique is used in those applications where robots must do the activities in a closed environment such as in industry. On the other hand in the local path planning techniques information of the environment is taken with the help of the sensors and executed during the movements of the robots. Local path planning techniques are mostly used in those robots where robots are required to perform random activities.

In this research a unified path planing technique is proposed which uses a combined global and local path planning method, in such a way the robot follows shortest path to a goal by avoiding obstacle. Initially, when both initial and goal positions are known, the environment of path planning is scanned, and all the static obstacles are mapped, by using global path planning. In the case if some dynamic obstacle or unknown static obstacle suddenly appears local path planning will be used. For global path planning A* algorithm is used while for local path planning Velocity Obstacle algorithm is used.

#### 2.2.1 A^⁎^ algorithm

The A* algorithm is one of the simple and optimized path planning technique which is normally used in mobile robot in known environment. A* algorithm is a cell (grid) base algorithm. The complete environment is divided in equal size of cells. Value of each cell is based on the cost and heuristic value of each cell. The cost of each cell is based on the distance of each cell from the start point and heuristic value of each cell is based on the distance of each cell from the goal point. In A* algorithm the shortest path is calculated by computing the value of each cell. Only those cells are selected whose values are lower than other cell values.

At each step, the value of *n* is selected based on values of the function f(n) in Eq. (10) as;

(10) f(n)=g(n)+h(n)

Where g(n) is the cost of each cell from the start node, and h(n) is the heuristic value of each node from the goal point. To implement the A* algorithm the whole environment is selected in which robot has to be moved to perform different activities. The complete information of obstacles is noted before moving the robot [29].

#### 2.2.2 Velocity obstacle algorithm

Velocity obstacle algorithm is used to avoid the dynamic obstacles. The dynamic obstacles are those which are not considered while implementing A* algorithm. The dynamic obstacles could be statics or moving obstacles. [17]. Two circular objects $r_{o}$ and $o_{b}$ are considered, where $r_{o}$ represents the mobile robot and $o_{b}$ is considered as a dynamic obstacle. Mobile robot has radius of $R_{ro}$ and obstacle has radius of $R_{ob}$. In velocity obstacle algorithm, mobile robot $r_{o}$ is reduced to be point robot and obstacle $o_{b}$ is enlarged by combining the radius of mobile robot and obstacle $\left(R_{ro}+R_{ob}\right)$. Collision cone is defined as all sets of relative velocities whose intersection with the obstacle is null or empty set. Collision cone can be described as follows;

(11) $$CC_{ro,ob}=\{v_{ro,ob}\|\lambda_{ro,ob}\cap R\neq \phi \}$$

where $CC_{(}ro,ob)$ is described as collision cone, $v_{ro,ob}$ is the relative velocity vector, $\lambda_{\left(ro,ob\right)}$ are the projection of relative velocity $v_{\left(ro,ob\right)}$ and R are the radius of enlarged obstacles which is $\left(R_{ro}+R_{ob}\right)$.

The collision cone is firstly computed by calculating, the distance between point robot and obstacle. Then angle *θ* between obstacle and point robot is calculated. After calculating angle *θ*, both minimum and maximum angle of collision cone (θ−β,θ+β) are calculated. Then both projected line $\lambda_{f}$ and $\lambda_{r}$ are calculated in such a way that both lines intersect with the obstacle.

**Figure 3:**
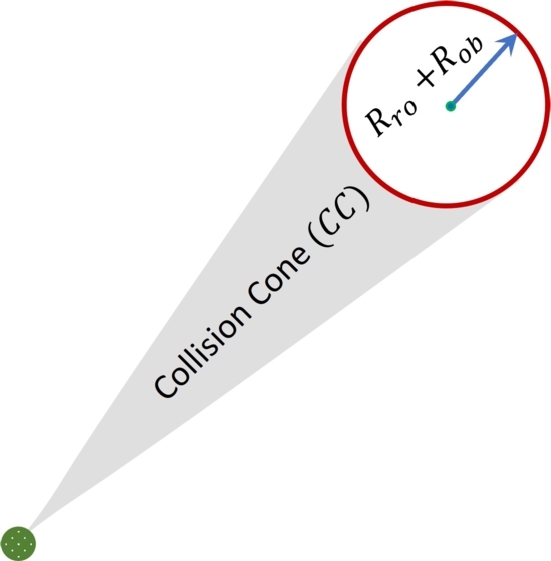
Collision cone between point robot and obstacle.

The collision cone between point robot and obstacle is shown in Fig. 3. Relative velocity vector $v_{\left(ro,ob\right)}$ is calculated based on the velocity of point robot and obstacle. This relative velocity vector is extended by $\lambda_{\left(ro,ob\right)}$ in such a way that it intersects with obstacle. It means any relative velocity vector which is in the range of $\lambda_{f}$ and $\lambda_{r}$ will cause the collision. Collision cone is specific with only one obstacle at a time. Since collision cone is usually used for static obstacles only, for moving obstacles, collision cone is transformed into velocity obstacle by adding the obstacle's velocity $v_{ob}$ to each set of collision cone. Makowski sum is used to add the scalar quantity to a vector quantity. Velocity obstacle is described in following equation;

(12) $$VO=CC_{\left(ro,ob\right)}⊕v_{ob}$$

Each obstacle has its own velocity obstacle. For multiple obstacles, velocity obstacle of every obstacle is computed and then following equation is used to calculate the VO by equation;

(13) $$VO=U_{i=1}^{k}VO_{ob_{i}}$$

Where k is the number of obstacles. In velocity obstacle algorithm, time horizon is used to avoid the complexity of algorithm. Time horizon is any random time value which is selected. After selecting that time, velocity of every obstacle is already known or measurable. So, from velocity and time, distance is calculated. In that time horizon only, those obstacles are considered whose distance from point robot is less than that distance. Time horizon can be calculated as follows;

(14) $$VO_{h}=\{V_{r}o\in VO_{h}||v||d/t_{h}\}$$

Where $t_{h}$ is the time horizon, d is the distance between robot and obstacle and $VO_{h}$ is the velocity obstacle in that time horizon.

**Figure 4:**
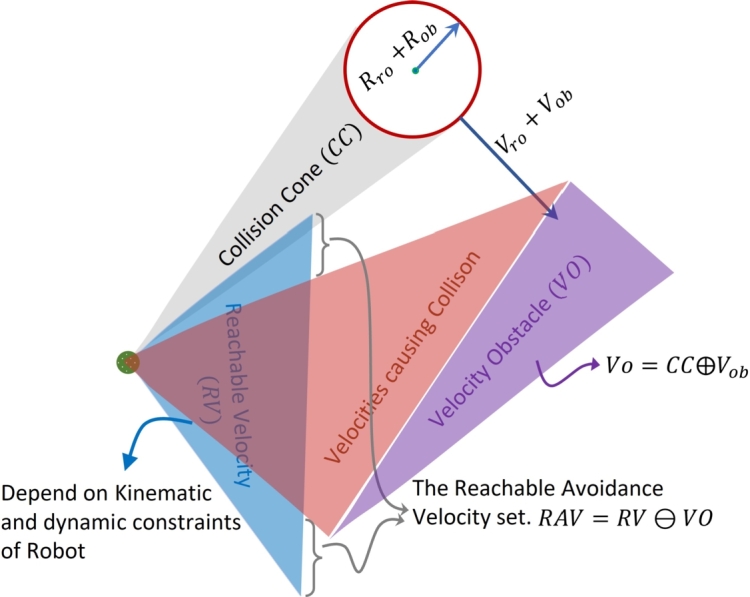
Collision cone for reachable velocity.

The detailed illustration of collision cone, velocity obstacle, reachable velocity and reachable avoidance velocities cones is shown in Fig. 4. Here the main factor in the velocity of the robot is adjustment of feasible accelerations. The feasible acceleration of mobile robot depends on its dynamics and its actuator constraints. Actuator constraints means the maximum and minimum velocity and acceleration of mobile robot. This set of feasible acceleration is multiplied with every instant of time and then added with mobile robot's velocity. Reachable velocity of mobile robot can be calculated by using following equation;

(15) $$RV(t+\delta t)=v|v=v_{ro}(t)⊕\delta tFA(t)$$

Where $v_{ro}$ is the velocity of mobile robot. FA(t) is the set of feasible acceleration.

Reachable avoidance velocity of mobile robot is the set of those velocities which ensures the collision free trajectory for mobile robot as shown in Fig. 4. The reachable avoidance velocity is computed using the following equation;

(16) RAV(t+δt)=RV(t+δt)⊖VO

#### 2.2.3 Proposed robot control algorithm

The control algorithm is so designed that a hybrid A* and velocity obstacle algorithm is used for motion control of the mobile robot. Initially, the start point, goal point, desired velocity, and threshold distance are defined. Afterward, the A* algorithm is employed to determine the shortest path, and the robot initiates its trajectory along the computed path. In order to keep the robot on calculated path, the required RPM for each wheel is calculated using Eq. (8), continuously. Each wheel is actuated by a separate motor which have much higher torque limit that it required to move the mobile robot. Hence, the motor was perfectly achieving the giving RPMs in order to keep the robot at calculated path. The actual velocity of the robot in terms of components is also validated using the Lidar sensor such that the robot perfectly achieves the commanded velocity.

**Figure 5:**
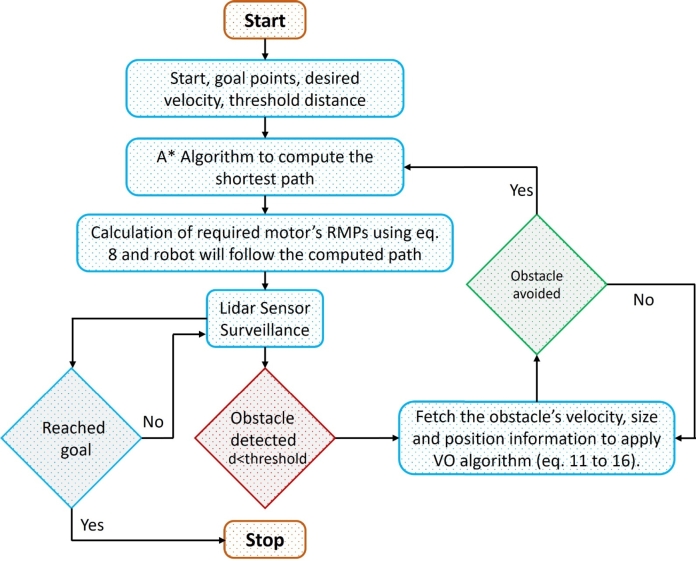
Flowchart of hybrid A* and velocity obstacle control algorithm of mobile robot.

In addition, in case of obstacle is detected and the current distance is less than the threshold distance then velocity obstacle algorithm is implemented as given in Eq. (11) and (16). The same algorithm and control scheme is applied in each experiment; however, the start point, goal point, desired velocity threshold distance related information was changed. A detailed flowchart of the proposed path planning and obstacle avoidance control algorithm is shown in Fig. 5.

### 2.3 Experimental setup

#### 2.3.1 Mobile robot

**Figure 6:**
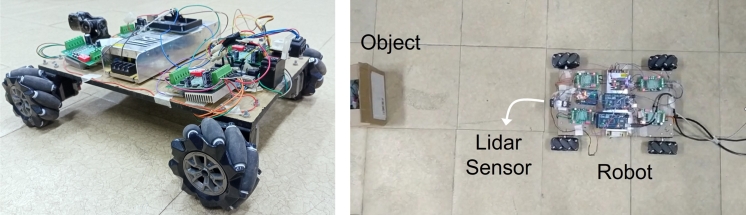
Experimental setup, robot with mecanum wheel and obstacle.

A mecanum wheel based mobile robot is designed to implement the proposed path planning algorithm as shown in Fig. 6. Four NEMA-17 stepper motors are used as actuators for menacum wheels. In order to control the direction and speed of stepper motors, TB 606516 motor drivers are used for each wheel. Wheels are one of the most important components in mobile robot. Mecanum wheels are used in this experimental setup. To implement the algorithm Arduino Mega 2560 is used as micro-controller. Lidar TF mini is used for obstacle detection and distance calculation. Lidar sensor is installed on a servo motor to scan the front environment of the mobile robot. Arduino Uno is used to control the servo motor and run the Lidar sensor. Servo motor rotates from -30 to 30 degree to scan the environment.

#### 2.3.2 Lidar sensor noise filter

Lidar sensor is used to measure the distance of the robot from any obstacle and to calculate size, shape and velocity of the obstacle. In real time applications, sensor output is not according to actual value due to number of reasons such as time lag, response, and noise. A lot of filters are used to reduce the noise. In this research, obstacle's size and shape is calculated and validated with the actual value and percentage difference is calculated and percentage difference is less than 10% which is acceptable. However, it can be further improved by the noise removal filter such as Exponentially Weighted Moving Average (EWMA) filter. The output can be calculated using following equation;

(17) $$Y_{t}=\alpha (X_{t}+(1-\alpha )⁎X_{(}t-1)+{\left(1-\alpha \right)}^{2}⁎X_{(}t-2)+{\left(1-\alpha \right)}^{3}⁎X_{(}t-3))p+q$$

Where $Y_{t}$ is the output from the filter, $X_{t}$ is current sensor value, and $X_{(}t-1)$ is the previous values of the sensor. By using the iteration of Eq. (17), noise is reduced, value of *α* can be selected between 0 and 1. At 0 value there is no filter and the raw data is coming from the sensor. The higher the value of *α* the most values are filtered out. The value of *α* can be selected according to the requirements and sensor values.

#### 2.3.3 Obstacle size

**Figure 7:**
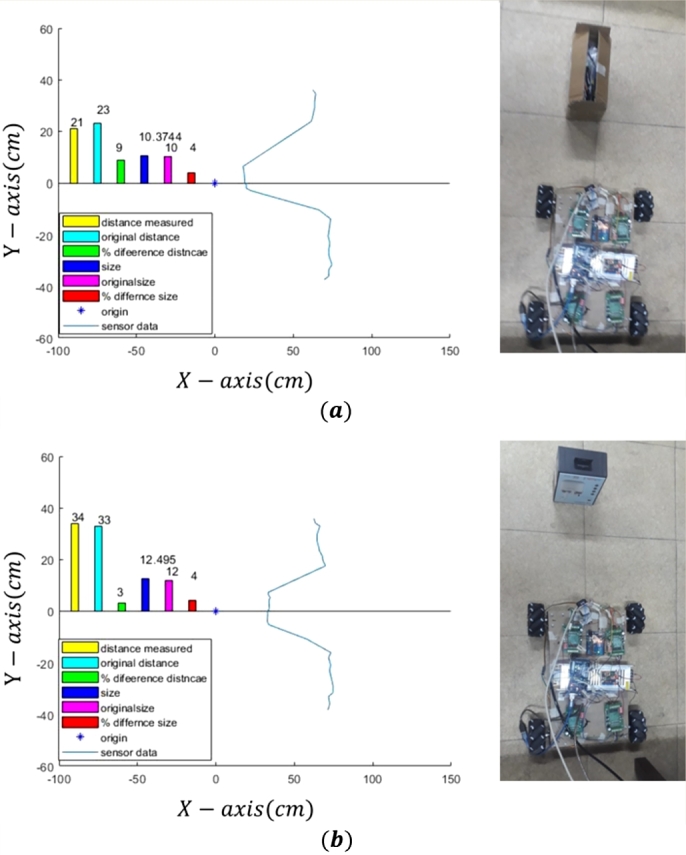
Obstacle size calculated using the Lidar Sensor; a) small size, and b) larger size obstacle.

This section describes the size of obstacle measure using the Lidar sensor. Lidar sensor is installed on the servo motor to scan the environment. Servo motor rotates from -30 to 30 degree to scan the environment. The obstacle is detected and its size is calculated through the Lidar sensor. Fig. 7 describes the obstacle size which are calculated using the Lidar Sensor. This size of obstacle is compared with the actual size and percentage error is calculated. This difference is 4% due to the noise in data values and this noise can be removed using different low pass filters. In Fig. 7(a) object of smaller size is taken while in Fig. 7(b) object of larger size is taken. It can be observed that the estimated size is different for both the cases.

## 3 Results

### 3.1 Simulation results

Initially the validation of proposed algorithm is checked using Matlab simulation. Various simulations with static obstacle only and then dynamic obstacles are performed, where the proposed algorithm is implemented in a closed environment. Path is computed according to the application of the mobile robot. Grid environment is made in such a way that the length and width of the environment is computed and start point of the robot which is (30, 15) and goal point which is (33,59) mentioned in the MATLAB such that it searches all the nearest cell and only computes those cells who have low cost (distance). The velocity of mobile robot is 1 m/s. Sampling time of 1 ms is used for simulation results which is also used during experiments, considering the specification of onboard computer Arduino.

**Figure 8:**
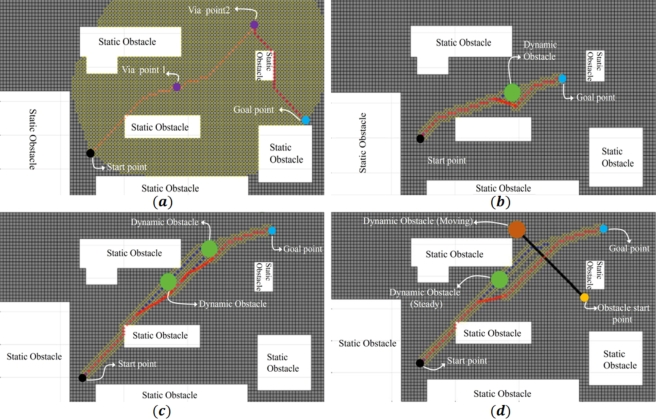
Simulation results for path planing of mobile robot; (a) using via point through A* obstacle, (b) avoiding static and one dynamic (steady) obstacle, (c) avoiding static and one dynamic (steady) obstacle, and (d) in dynamic environment.

In this section mobile robot must reach its destination by some via points. The first via point is the initial goal point of the mobile robot after reaching this point after that this via point will be the start point for the mobile robot and next via point is the goal point. Next start point is this via point and goal point is the final goal point. Simulation result of the path planning is shown in Fig. 8(a).

In Fig. 8(b), the simulation results of path planning of mobile robot with static obstacles and one dynamic obstacle is shown. The starting point of the mobile robot (30,21) and goal point is (70,36) mentioned. The velocity of mobile robot is 1 m/s. Shortest path for the mobile robot is selected using A* algorithm. Position of the dynamic obstacle is known and distance from robot to obstacle is calculated and when distance is less than a threshold which is 20 cm velocity obstacle is implemented to avoid the obstacle and again the A* algorithm is implemented to find the shortest path to reach the goal point.

In Fig. 8(c), simulation is done with two dynamic (steady) obstacles and various static obstacles are considered in the environment. Both start and goal point are known so they used to find the shortest path using A* algorithm. Velocity of mobile robot is 1 m/s. Dynamic obstacles are placed randomly in the environment. Positions of the obstacles are known or measurable so distance of obstacle to robot is computed continuously. When the distance is less than threshold which is 20 cm then velocity obstacle algorithm is implemented to avoid the obstacle and A* algorithm is implemented to find the shortest path to reach the goal position and again distance to obstacles computed. When distance is again less than threshold velocity obstacle algorithm is implemented to avoid the obstacle. The A* algorithm is implemented to reach the goal position once again.

In Fig. 8(d), the simulation results of path planning in dynamic environment is given, where both static and dynamic obstacles are placed. The Starting and goal point of mobile robot are known, and shortest path is calculated by applying the A* algorithm. The position and velocity of dynamic obstacles are known and distance from robot to obstacles are calculated. Velocity of mobile robot is 1 m/s. When distance is less than a threshold value, velocity obstacle is implemented to avoid the dynamic obstacle. After that the A* is again implemented to find the shortest after the obstacle avoidance.

### 3.2 Experimental results

Real time experimentations are performed to validate the proposed path planning algorithm, formed by combination of A* and velocity obstacle algorithms. In velocity obstacle algorithm, position of mobile robot, its velocity, positions and velocities of obstacles are either known or measurable. To validate the dynamic obstacles, mobile robots position is calculated at every point, obstacle size and position are calculated through the Lidar sensor. Lidar sensor is mounted on robot to determines the distance between obstacle and mobile robot. This distance is converted into its components which describes the position and size of obstacle. The velocity obstacle algorithm is implemented on different environments. Firstly, it is applied on the single static obstacle followed by multiple static obstacles and lastly it is implemented on the dynamic obstacle.

**Figure 9:**
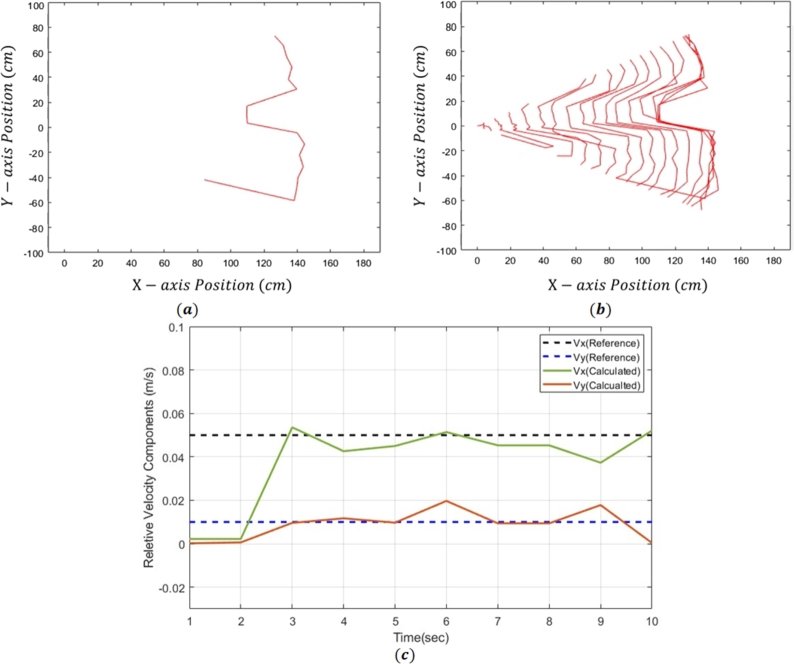
(a) Distance of obstacle from starting point, (b) distance goal relative to obstacle, and (c) velocity of mobile robot.

In Fig. 9(a), the distance of obstacle is given using the Lidar sensor, while in Fig. 9(b), the distance of goal relative to obstacles using Lidar sensor is given. The actual and estimated velocity of the robot is calculated experimentally taking the derivative of distance covered by the robot. It can be observed in Fig. 9(c), that the actual velocity and calculated velocity are almost same. This size of obstacle, calculated using Lidar sensor, as already given in Fig. 7.

**Figure 10:**
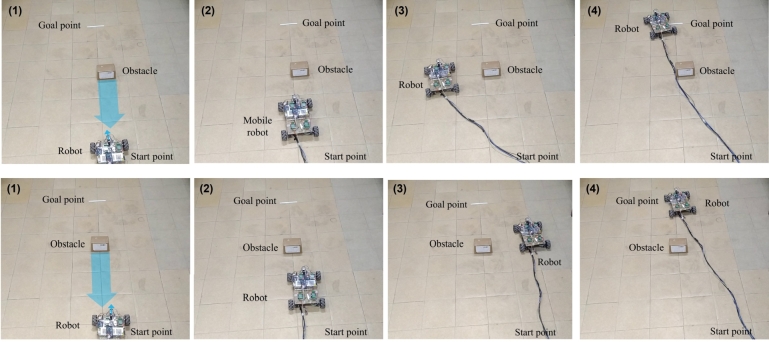
Sequential snapshot of motion of robot with one static obstacle, using proposed algorithm.

In Fig. 10, a sequential snapshot of motion of robot in presence of a single static obstacle is given. The starting point of mobile robot is considered is (0,0) and goal position of the robot taken as (2,0) meter. The threshold distance to use velocity obstacle is set according to the size of the obstacle. It can be observed in Fig. 10 that the mobile robot follows the goal points until threshold reached, where the threshold collision cone is calculated. After the collision cone is originated, the set of relative velocity is calculated based on the two angles of the collision cone and checked if the relative velocity of the mobile robot is in that set which will lead to collision. So those angles are considered to calculate the mobile robots velocity which are not in collision cone when the obstacles are avoided again robots current position and the goal points are noted and angle from robots current to the goal points is calculated, to reach the goal position. When the distance between robot and obstacle is equal to the threshold distance, velocity obstacle algorithm is implemented. The threshold value is set to 60 cm distance. When the distance is less than 60 cm velocity obstacle algorithm is implemented. At the distance of 60 cm mobile robots position is noted which is 30 cm on X-axis and 0 cm in Y-axis. While the obstacle's position is estimated which is (60,-8) cm in left side and (60,10) cm in right side. The mobile robot moves with that angle until it avoids the obstacle. After the robot moves with -50 degree to avoid the obstacles. Robot will follow the same velocity until it avoids the obstacle completely. Mobile robots position is noted at every iteration and when mobile robot's position is greater than obstacle it calculates the angle from robots current point to the goal point again using A* mobile robots moves with that computed velocity until it reached to the goal point.

**Figure 11:**
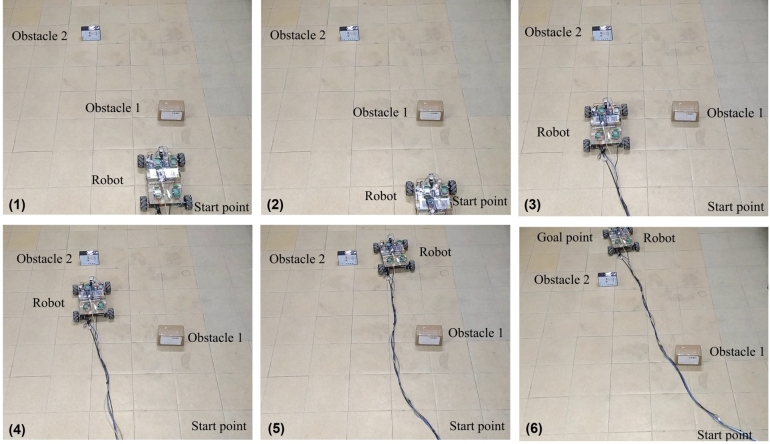
Sequential snapshot of motion of robot with two static obstacles, using proposed algorithm.

The sequential snapshots of the experiments are shown in Fig. 11 where proposed algorithm is also applied for two static obstacles. The robot's starting and goal positions are known and robot's velocity is determined by calculating the angle from start to goal position. The starting point of mobile robot is selected as (0,0). It can be observed that the robot moves towards the goal while avoiding both the obstacles using velocity obstacle. The threshold distance in this case is considered as 40 cm.

**Figure 12:**
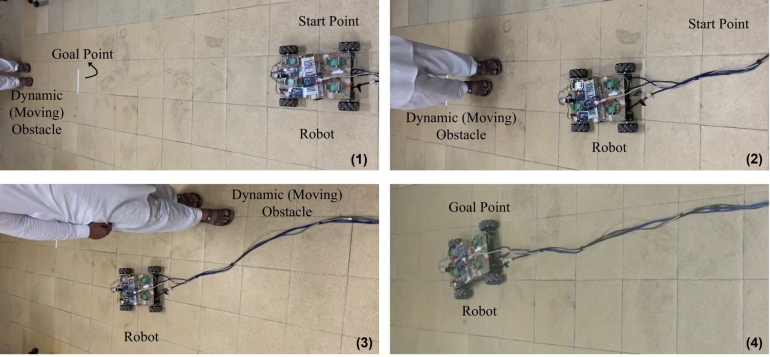
Sequential snapshot of motion of robot with a dynamic obstacle, using proposed algorithm.

In Fig. 12 the sequential snapshots of proposed path planing algorithm applied on dynamic obstacle are given. A person moving in the workspace is considered as a dynamic obstacle. Obstacle's position and size is calculated through the Lidar sensor. Robot's starting and goal positions are known and starting position is considered as (0,0), dynamic obstacle and the robot goal point which is (2.5,0) meter relative to start point. A threshold value selected to be 40 cm, set according to the size of the obstacle. It can be observed that the mobile robot moves towards the goal points until threshold reached after the threshold robot position and obstacle position are known and collision cone is formed. After the collision cone is originated, velocity obstacle is calculated. The velocity obstacle, avoidance angle is calculated if mobile robot moves with a velocity which lies in velocity obstacle region will lead to the collision. So those angles are selected which are not in the collision cone region. When the obstacles are avoided again robot's current position and the goal points are again noted and angle from robot's current to the goal points is calculated. Velocity components of mobile robot are calculated based on that angle. Mobile robot's move with that velocity until it reached to the goal point.

### 3.3 Proposed application

**Figure 13:**
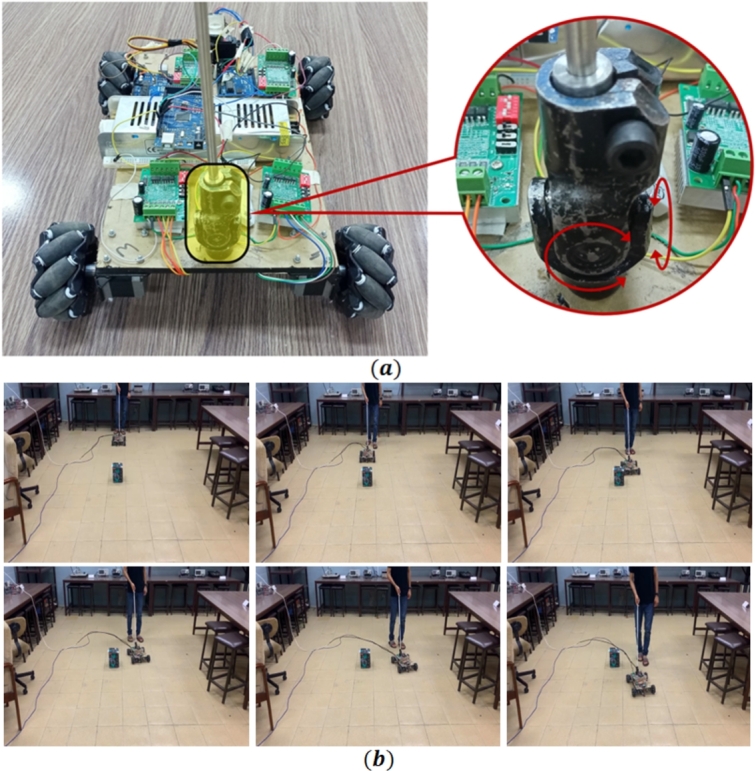
Application of mobile robot as an assistive robot for visually impaired person; (a) attachment of white cane to the robot using universal joint, (b) movement of white cane attached to robot in structured environment.

It can be observed through experimental results that the robot easily avoids static and dynamic obstacles using the proposed algorithm. Therefore, it can be used in various applications for human benefit. In Fig. 13 (a) the robot is modified as an assistive device for visual impaired people where a white cane is attached to the robot through a universal joint. A visually impaired person can easily move using the cane connected with the mobile robot as shown in Fig. 13 (b). Using this application as assistive device a visually impaired person can move around freely while avoiding various obstacles in the way.

## 4 Conclusion

A strategy to design and path planning of mecanum wheel based mobile robot is proposed and implemented in this paper. The advantage of the mecanum wheels based mobile robot is that it can move in any direction without turning which is very useful in congested environment. The proposed path planing algorithm is combination of an A* algorithm and a velocity obstacle algorithm in an environment with both static and dynamic obstacles. For the shortest path planning, the A* algorithm is utilized, while avoiding the static obstacles. However, in real time applications the challenge is to avoid the dynamic obstacles, for this purpose the velocity obstacle algorithm is combined with A* algorithm for autonomous navigation and path planning of mobile robot. To validate the proposed algorithm both simulation and experiments are used and it was observed the proposed method helps the robot to follow the shortest path while avoiding the static and dynamic obstacles.

A qualitative comparative analysis is performed between the proposed work and recently published research activities as shown in Table 1. The implementation of the velocity obstacle method in real-time is challenging as the obstacle size, position, and velocity vector need to be calculated in real time. The A* algorithm alone provides the optimized path in a static environment only. Some researchers used ultrasonic sensors which have a lower range and data acquisition speed compared to lidar sensors as shown in Table 1. In comparison, the proposed work clearly extends the research work related to real-time path planning in a dynamic environment with the following integrated capabilities;

– Multiple static and dynamic obstacle avoidance.
– Single local sensor to avoid dynamic obstacles.
– Extension and real-time application related to the assistance of visually impaired people.
– Used mecanum wheel for sharp sideways motions to avoid the fast-moving dynamic obstacle, particularly in small spaces.
– Possible application of proposed robot and algorithm, which in this case is an assistive device for visually impaired person.

**Table 1:** Feature comparison between previous research and proposed algorithm for mobile robot with mecanum wheels.

Reference	Sensor Used	Obstacles	Environment	Algorithm Used	Application
[30]	Local/Low-range	Multiple	Statics	Artificial bee colony optimization	Not provided
[31]	Local lidar	Multiple	Static	Hybrid A*, PRM, RRT, and RRT*	General
[32]	Local lidar	Multiple	Static	Improved A* algorithm	Provided
[33]	Simulation	Multiple	Static & dynamic	Artificial potential field and SMC	Not provided
[34]	Multiple (local)	Multiple	Static & dynamic	Improved A* & dynamic window	General
[35]	Multiple (local)	Single	Static & dynamic	Waypoint Navigation	General
Proposed	Local lidar	Multiple	Static & dynamic	Unified A* & Velocity obstacle Alg.	Provided

## Funding

This work was supported by Higher Education Commission, Pakistan (HEC-NRPU-14813 and HEC-TDF-02-203). Author Abid Imran and Sajjad Manzoor have received research supports from Higher Education Commission, Pakistan.

## Consent to participate

Informed consent was obtained from all individual participants included in the study.

## Code availability

Can be provided on request.

## CRediT authorship contribution statement

**Muhammad Umair Shafiq:** Writing – original draft, Validation, Methodology. **Abid Imran:** Writing – original draft, Software, Methodology. **Sajjad Maznoor:** Writing – review & editing, Software, Investigation. **Afraz Hussain Majeed:** Writing – review & editing, Resources, Conceptualization. **Bilal Ahmed:** Writing – review & editing, Methodology, Investigation. **Ilyas Khan:** Validation, Resources, Investigation. **Abdullah Mohamed:** Visualization, Validation, Resources, Conceptualization.

## Declaration of Competing Interest

The authors declare the following financial interests/personal relationships which may be considered as potential competing interests: Sajjad Manzooe reports equipment, drugs, or supplies was provided by Higher Education Commission, Pakistan. If there are other authors, they declare that they have no known competing financial interests or personal relationships that could have appeared to influence the work reported in this paper.

## Data Availability

Data can be provided on demand.
